# Isolation of the Main Biologically Active Substances and Phytochemical Analysis of *Ginkgo biloba* Callus Culture Extracts

**DOI:** 10.3390/molecules28041560

**Published:** 2023-02-06

**Authors:** Violeta Le, Andrey Sukhikh, Timothy Larichev, Svetlana Ivanova, Alexander Prosekov, Anastasia Dmitrieva

**Affiliations:** 1 Natural Nutraceutical Biotesting Laboratory, Kemerovo State University, Kemerovo 650043, Russia; 2 Laboratory of Physico-Chemical Studies of Pharmacologically Active and Natural Compounds, Kemerovo State University, Kemerovo 650043, Russia; 3 Department of Fundamental and Applied Chemistry, Kemerovo State University, Kemerovo 650043, Russia; 4 Department of General Mathematics and Informatics, Kemerovo State University, Kemerovo 650043, Russia; 5 Laboratory of Biocatalysis, Kemerovo State University, Kemerovo 650043, Russia

**Keywords:** *Ginkgo biloba*, callus cultures, biologically active substances, highly effective chromatography, antioxidant activity, geroprotective properties

## Abstract

The work reveals the results of studying the content of biologically active substances in samples of extracts of *Ginkgo biloba* callus cultures. Callus cultures grown in vitro on liquid nutrient media were the objects of the study. Considering various factors affecting the yield of the target components during extraction, the volume fraction of the organic modifier in the extracting mixture, the temperature factor, and the exposure time were identified as the main ones. The maximum yield of extractive substances (target biologically active substances with a degree of extraction of at least 50%) from the samples of callus culture extracts was detected at a ratio of extragent of 70% ethanol, a temperature of 50 °C, and exposure time of 6 h. Flavonoids, such as luteolin, quercetin, isoramentin, kaempferol, and amentoflavone, were isolated in the extract samples. As a result of column chromatography, fractions of individual biologically active substances (bilobalide, ginkgolide A, B, and C) were determined. The proposed schemes are focused on preserving the nativity while ensuring maximum purification from associated (ballast) components. Sorbents (Sephadex LH-20, poly-amide, silica gel) were used in successive stages of chromatography with rechromatography. The degree of purity of individually isolated substances was at least 95%.

## 1. Introduction

*Ginkgo biloba* has been used for medical purposes since ancient times. It is the only surviving relict plant species of the *Ginkgoaceae* family of gymnosperms. *Ginkgo biloba* is characterized by the content of condensed tannins, terpene trilactones, flavon glycosides, organic acids, amino acids, and trace elements [1,2]. Plant components can exhibit various complementary pharmacological effects [3,4,5,6]. Many studies have been published on the qualitative and quantitative analysis of the components of *Ginkgo biloba* and the phytopreparations based upon it [7,8,9,10,11,12]. According to the standardization criteria, the content of the following main active components is determined: flavonoids, sesquiterpenes, and dominant diterpene lactones (ginkgolides) [13]. 

Diterpene glycosides have a variety of biological activities, which significantly expands the possibilities for creating new drugs based on their molecular complexes.

*Ginkgo biloba* is one of the most promising plants for the creation of drugs with neuroprotective effects (plant extracts are used to treat Alzheimer’s disease, Parkinson’s disease, cerebral vascular deficiency, and dementia) [14]; they offer the ability to protect the vascular endothelium, increase insulin resistance, and prevent atherosclerosis [15], and also have antitumor effects due to the ability of polyphenols to influence oxidative stress [16]. They have also previously been reported to possess antiparasitic, antifungal, antibacterial, and antiviral activity [17].

Limited resources and high demand for the raw material base of *Ginkgo biloba* for the pharmaceutical industry dictate the need to search for and develop alternative sources for obtaining valuable pharmacotherapeutic extracts [18]. The technology of cultivation of plant cells and tissues is a possible tool for studying both the process of biosynthesis and the production of the secondary metabolites of plants [19]. On the other hand, obtaining individual substances produced by the *Ginkgo* callus culture is important for basic research, as well as for the above-mentioned applications. The aim of the study is to evaluate the effectiveness of the extraction technique and the preparative accumulation of key components produced by the *Ginkgo biloba* callus culture. The novelty of the study lies in the development of methods for obtaining targeted biologically active substances, which is a strategically significant priority task. At the present stage, callus cultures are considered as an alternative for the production of plant biologically active ingredients [20]. In the proposed study, extracts containing biologically active substances were isolated from *Ginkgo biloba* callus cultures. The technological modes providing the high degree of extractive substances are investigated. When performing phytochemical analysis using spectral and chromatographic methods, the component composition of extracts of *Ginkgo* callus cultures was studied. An algorithm for the selection of target components has been proposed that allows scaling the process during the preparatory isolation of the key components of the extract. 

To date, considerable attention has been paid to the aspects of extraction from the *Ginkgo biloba* phyto-object. This circumstance is connected with the need to ensure the completeness of extraction of target biologically active substances from a plant source by using cost-effective and environmentally friendly extractants [21,22] and reducing the content of ballast substances released simultaneously, including toxic components, such as 4-O-methylpyridoxine [23,24]. In this regard, special attention should be paid to the studies aimed at developing effective methods for obtaining standardized extracts or individual components intended for use in the food and/or pharmaceutical industries [25,26,27].

In one of the studies [28], the possibility of increasing the efficiency of extraction of terpene trilactones from *Ginkgo biloba* leaves using ultrasound and deep eutectic solvents (mixtures of choline with urea and betaine with ethylene glycol) was proposed. These solvents gave higher extraction yields than the well-known, most effective solvent, namely 70% ethanol. The extraction conditions were as follows: a mixture containing 40% (wt./wt.) water was used as an extraction solvent, with a 1:10 ratio of *G. biloba* leaf powder to solvent, and ultrasonic treatment was performed at 45 °C and 100 W for 20 min. The total extraction yield (1.94 ± 0.03 mg/g) was obtained under optimal conditions, indicating that 99.37% of triterpene lactones can be extracted from *G. biloba* leaf powder by a single extraction. For purification, the authors used a polyamide sorbent and achieved an extraction yield of 95.1%. However, the pronounced potential nephrotoxicity of ethylene glycol does not allow it to be used for the manufacture of medicinal extracts. At the same time, unfortunately, the authors did not consider the effect of the eutectic mixtures used on the ability to extract accompanying polyphenolic compounds, and, in particular, flavonoids. In another original study, a cellulose-based eutectic natural polymer was used to isolate rutin from *Ginkgo* [29]. At the same time, the authors have shown the effectiveness of methanol for pre-extraction of the terpenoid fraction with subsequent pre-extraction with ethanol at temperatures above 70 °C, which requires compliance with the conditions associated with reducing solvent losses. At the same time, effective separation was achieved by using octadecyl-functionalized silica gel sorbent in the HPLC mode, which significantly limits the implementation of the preparative regime for the isolation and purification of individual pharmacologically active compounds from the *Ginkgo biloba* phyto-object. Furthermore, a dynamically developing area is the application of supercritical fluid extraction using such extractants as liquefied carbon dioxide. 

The features discussed above dictate the need to develop effective methods for obtaining standardized extracts and/or high-purity individual substances. The given paper reviews the studies on the production of highly purified components from total ethanol extracts of *Ginkgo biloba* callus cultures.

## 2. Results

For the analysis of the component composition of water–alcohol extracts of *Ginkgo biloba* (*Ginkgo biloba* (L.), fam. *Ginkgoaceae*), the spectrum of its native water–alcohol extraction was captured. It contained a plateau of about 270–275 nm, which was determined by secondary metabolites containing an aromatic hydroxyl group. Data on optical densities of Ginkgo biloba callus culture extract samples are presented in Table 1.

When assessing the influence of variable parameters on the extractivity of the main biologically active substances (BAS), it was found that the limiting factor contributing to maximum extraction was the concentration of the organic component and the extraction temperature. In [30,31,32], ethanol extraction is considered as the primary stage of isolation of the target components of the extract. At the same time, it is well known that the use of low molecular weight alcohols is the basis for isolating components of the flavonoid structure when obtaining total extracts from medicinal plant raw materials in the processing conditions of the chemical and pharmaceutical industry. This property of ethanol as a component approved for use in medical practice was an additional argument in favor of its use in the primary processing of callus culture and extract production.

**Figure 1:**
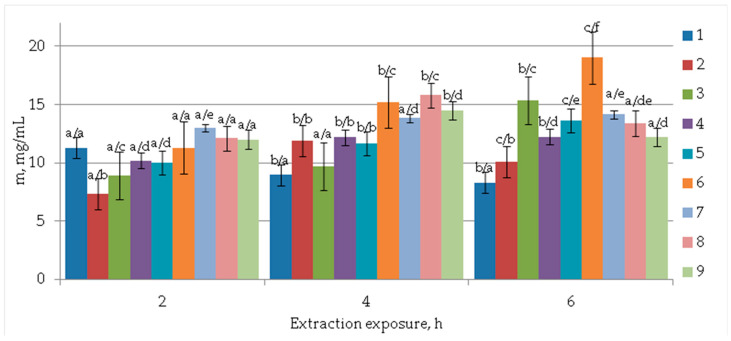
Effect of extractant concentration (C, %), extraction temperature (T, °C), and extraction exposure (τ, h) on extraction of target flavonoid components in terms of quantitative quercetin content (mg/mL), as follows: 1⁠—T = 30 °C; C = 30%, 2⁠—T = 30 °C; C = 50%, 3⁠—T = 30 °C; C = 70%, 4⁠—T = 50 °C; C = 30%, 5⁠—T = 50 °C; C = 50%, 6⁠—T = 50 °C; C = 70%, 7⁠—T = 70 °C; C = 30%, 8⁠—T = 70 °C; C = 50%, 9⁠—T = 70 °C; C = 70%. Data presented as a mean ±SD (*n* = 3). Values followed by the same letter do not differ significantly (*p* > 0.05) in extraction dynamics/in different extraction modes.

Initially, an extraction kinetic variant was used, which made it possible to determine narrower ranges and parameters for the variables studied in the work. A diagram of the dependence of the content of extractive flavonoids in terms of the quantitative content of quercetin is presented in Figure 1. When extracted with 70% ethanol at a temperature of 50 °C with an exposure time of 6 h, the target yield of the components in terms of quercetin was 19.02 mg/mL of the extract. Based on modern ideas about the component composition of ginkgo samples, the pool of flavonoids is represented by a variety of compounds. More than 50 flavonol glycosides and 7 flavonols are known [33]. Quercetin and its glycoside forms belong to one of the dominant compounds. Quercetin is used as a type-A standard marker for the standardization of pharmaceutically significant *Ginkgo biloba* preparations, and chromatographic approaches were proposed in USP 43- NF 38 [34]. Quercetin is a marker in the standardization of ginkgo preparations in the pharmacopeias of the USA, Great Britain, etc. Furthermore, our own research allows us to assert the dominant content of luteolin, quercetin, and their glycosidized forms in the studied samples of callus culture extracts.

**Figure 2:**
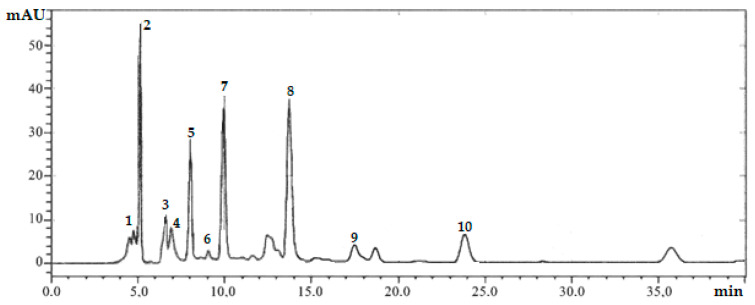
HPLC chromatogram of the extraction of *Ginkgo biloba*. Peak 1—ginkgolide A; peak 2— ginkgolide B; peak 3—bilobalide A; peak 4—ginkgolide C; peak 5—quercetin; peak 6—ginkgetin; peak 7—isorhamnetin; peak 8—luteolin; peak 9—kaempferol; peak 10—amentoflavone. The unnumbered peaks on the chromatogram belong to unidentified substances. Chromatographic conditions are as follows: mobile phase eluent A (water–acetonitrile 95:5 + 0.1% formic acid), mobile phase eluent B (acetonitrile–water 95:5 + 0.1% formic acid); flow rate 0.8 mL/min; column temperature 40 °C; injection volumes—20 µL; elution mode—gradient.

The extracts obtained with the maximum degree of substance content were analyzed by content for the main types of biologically active substances. In the studied samples of extracts, the presence of various groups of substances was determined using qualitative reactions. Reactions with ammonium hydroxide and the presence of fluorescence in UV light indicated the presence of flavonoids in the samples of *Ginkgo biloba* extracts [35]. Using chromatographic studies, five flavones were quantified in the samples, namely quercetin, isorhamnetin, luteolin, kaempferol, and biflavonoid amentoflavone. The presence of the largest number of flavonoids in the extract samples can be explained by the greater accessibility to the extraction of these substances under the applied extraction conditions [35]. The chromatogram of the samples is shown in Figure 2. The time parameters of retention on the column and the quantitative content are presented in Table 2. Ginkgolide A and ginkgolide B concentrations were not determined due to insufficient separation of these components during chromatography. A relatively high content was found for isorhamnetin and quercetin. Among ginkgolides, ginkgolide B was dominant, with a retention time of 5.16 min.

**Figure 3:**
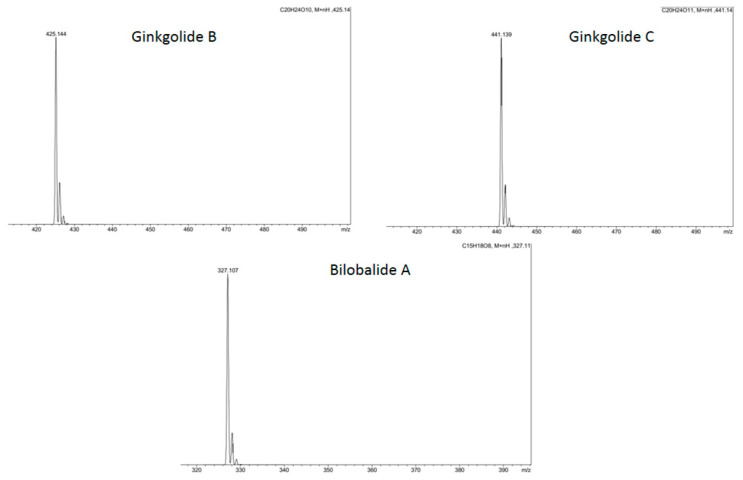
HRMS spectra of selected individual compounds.

To accurately determine the purity of the peaks of compounds 2, 3, 4 (ginkgolide B, ginkgolide C and bilobalide A), we obtained high-resolution mass spectrometry (HRMS) spectra. Here, HRMS spectrometry was performed using a Bruker Amazon speed ion trap in electrospray ionization (ESI) mode. The results of HRMS are shown in Figure 3. Peak purity control, as well as the identification of compounds by HRMS, allow for separation under conditions of acceptable resolution at the level of 10% between retention times. 

**Figure 4:**
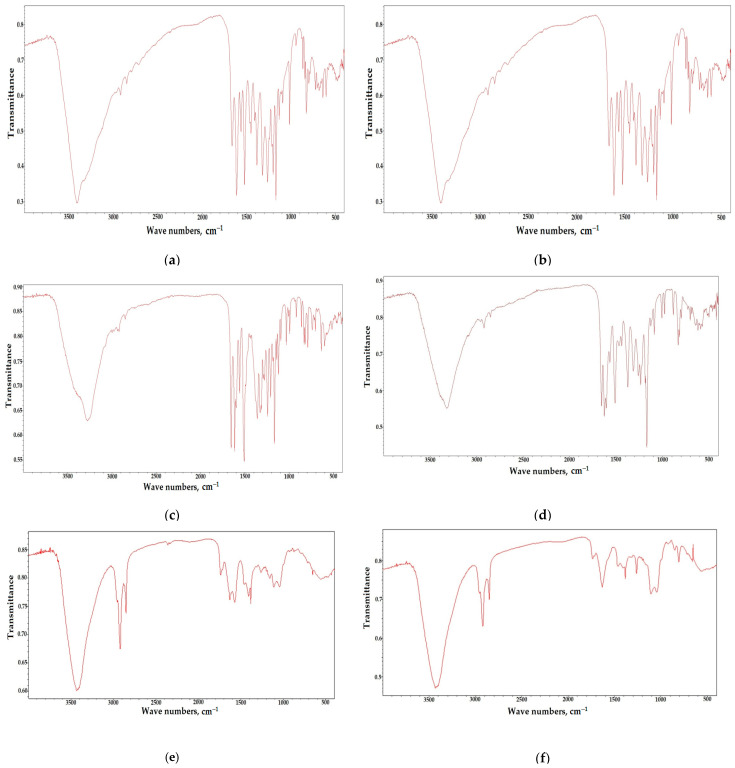

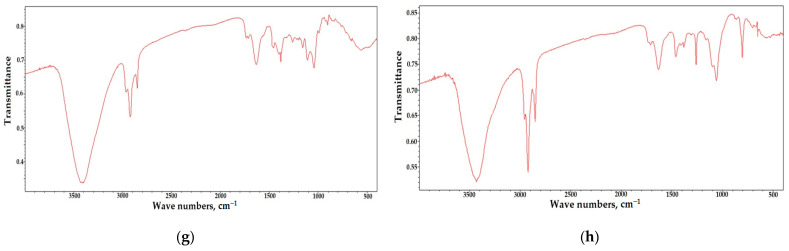
IR spectra of individual BAS. (**a**) Quercetin; (**b**) luteolin; (**c**) isorhamnetin; (**d**) kaempferol; (**e**) ginkgolide A; (**f**) ginkgolide B; (**g**) ginkgolide C; (**h**) bilobalide; with a degree of purification of at least 95% according to HPLC (wavelength 254 nm).

**Figure 5:**
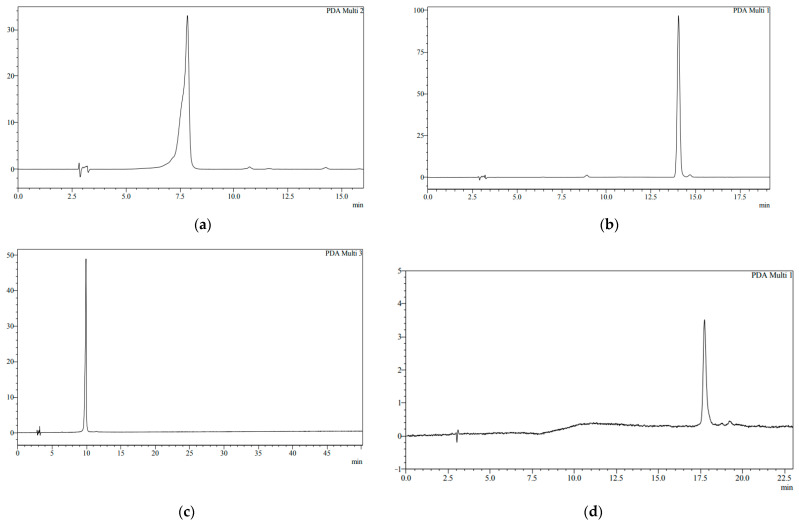

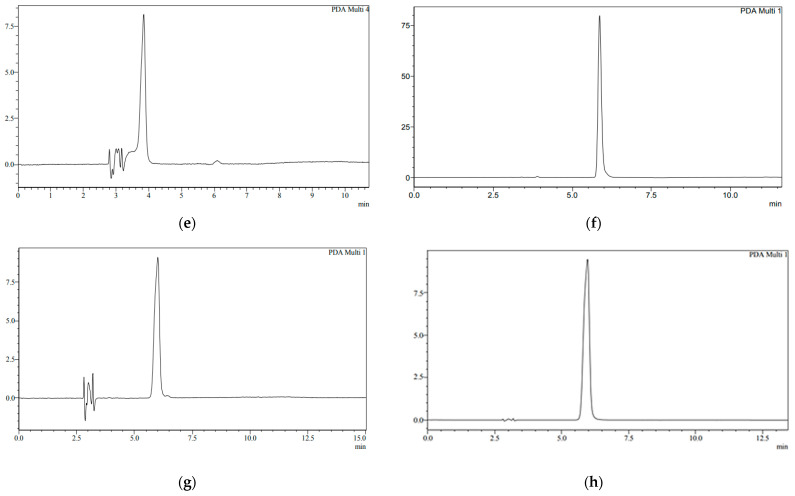
HPLC chromatograms of individual purified biologically active substances. (**a**) Quercetin; (**b**) luteolin; (**c**) isorhamnetin; (**d**) kaempferol; (**e**) ginkgolide A; (**f**) ginkgolide B; (**g**) ginkgolide C; (**h**) bilobalide A.

The main task in selecting rational parameters for the isolation of individual biologically active substances from extracts obtained from the biomass of *Ginkgo biloba* callus cultures was to isolate the sum of active substances in their native state, while ensuring adequate purification from accompanying (ballast) components. The results of the research allowed us to identify targeted biologically active substances with a degree of extraction of at least 50% from extracts of callus cultures (Table 3). The results of the purified samples of biologically active substances obtained are presented in Figure 4 by the IR spectrum of individual BAS with a degree of purification according to HPLC data of at least 95% (Figure 5). The given infrared spectra coincide with the absorption bands of the standards.

The IR spectrum of the quercetin flavone (3,5,7,3,4-pentaoxyflavone) was characterized by the following bands: an elongated band with an absorption maximum of 3405 cm^−1^ is specific to the 4-OH group of ring C; the presence of the 1661 cm^−1^ band is due to valence vibrations C = O; bands at 1611, 1561, and 1522 cm^−1^ are caused by C-C bonds of aromatic fragments of the quercetin molecule, rings A and B. In turn, the bands at 1460 and 1449 cm^−1^ are caused by plane vibrations C = C of the aromatic ring. The bands at 1407 and 1382 cm^−1^ are determined by plane deformation vibrations C-O-H and O-H, respectively. The band at 1166 cm^−1^ is caused by antisymmetric valence vibrations of C-O-C in the structure of the heterocycle. The presence of bands at 841, 824, 799, and 724 cm^−1^ is caused by out-of-plane deformation vibrations of the hydroxyl group of aromatic fragments of the B ring. At the same time, the hydrogen bond formed was traced at about 650 cm^−1^. The disubstituted ring B is characterized by the presence of an out-of-plane deformation vibration of the C-C bond at 702 cm^−1^.

The IR spectrum of kaempferol flavol (3,4,5,7-tetrahydroxyflavone) was characterized by the following distinctive features. The elongated band with an absorption maximum of 3322 cm^−1^ is specific for the 4-OH group of ring C. The presence of the band at 1657 cm^−1^ is due to valence vibrations C = O. The bands at 1612 and 1605 cm^−1^ are determined by the influence of the OH group at positions 3 and 5 of the heterocyclic fragment of the molecule, respectively, as a result of the formation of an intramolecular hydrogen bond with C = O. It was the influence of this hydroxyl that caused the distortion of the planar arrangement of the pyran fragment and the bond, and thereby led to the appearance of resonance.

The band of weak intensity at 1564 cm^−1^ is caused by C = C bonds of the aromatic systems of rings A and B. In turn, the bands at 1510 and 1467 cm^−1^ reflected the valence vibrations of the C-C bond of the aromatic ring. The band at 1372 cm^−1^ arose as a result of the interaction between deformation vibrations of O-H and valence vibrations of C-O in the structural components of kaempferol. The band at 1169 cm^−1^ is caused by antisymmetric valence vibrations of C-O-C in the heterocycle structure. The presence of the 831 cm^−1^ band is caused by out-of-plane deformation vibrations of the hydroxyl group of the B ring. The hydrogen bond formed was traced to about 650 cm^−1^. The mono-substituted ring B is characterized by the presence of an out-of-plane deformation vibration of the C-C bond at 702 cm^−1^.

The distinctive signals and band assignments for ginkgolides are presented in Table 4, Table 5, Table 6 and Table 7.

## 3. Discussion

**Figure 6:**
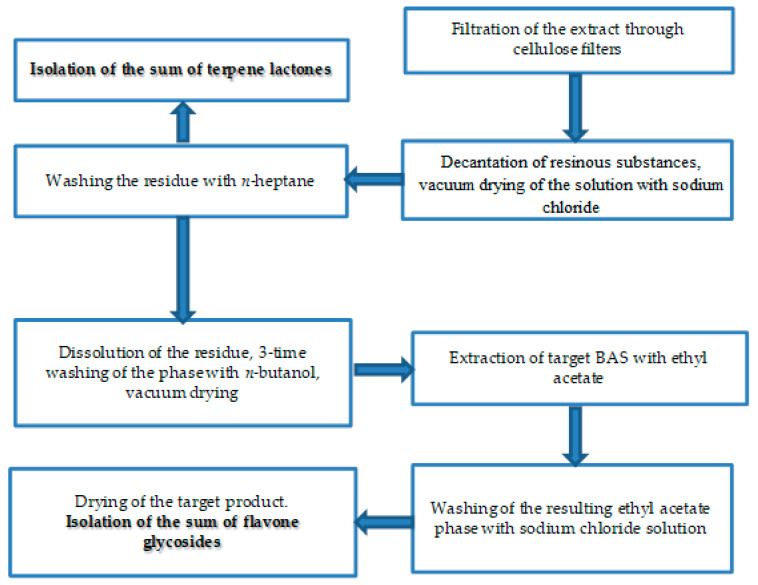
Scheme of isolation of biologically active substances from the extract of *Ginkgo biloba*.

**Figure 7:**
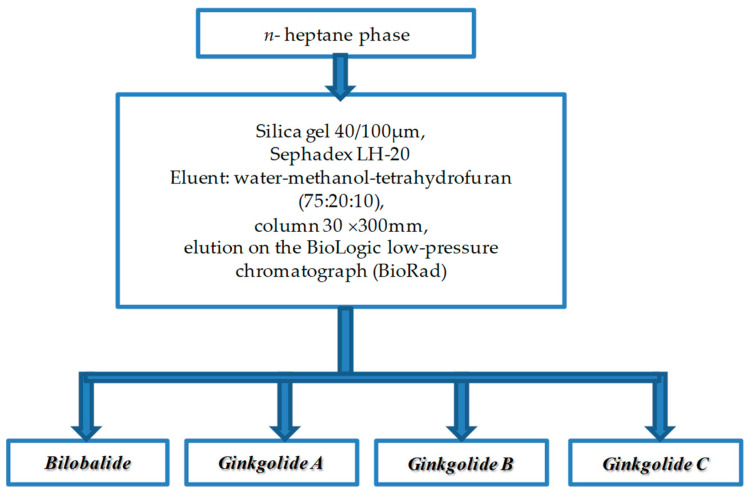
The developed scheme of purification of biologically active substances obtained from the *n*-heptane phase of *Ginkgo biloba* extract.

**Figure 8:**
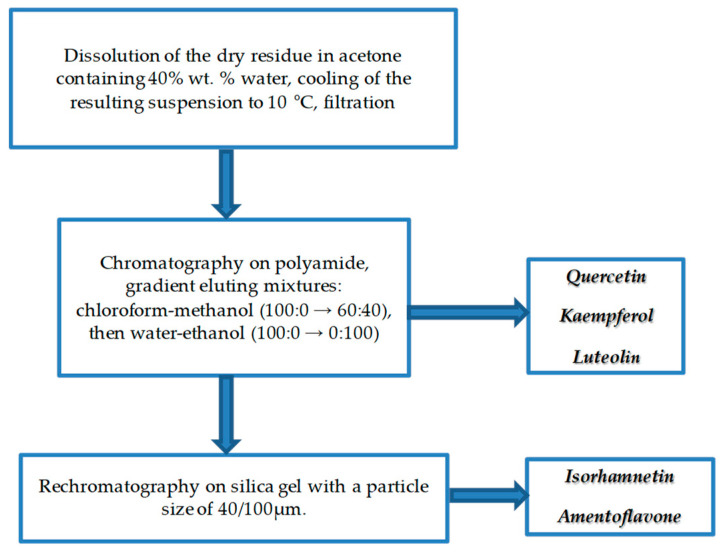
The developed scheme of purification of biologically active substances obtained from the extract of *Ginkgo biloba*.

When purifying individual biologically active substances obtained from the samples of extracts of *Ginkgo biloba* callus, adequate purification of the target components from accompanying ballast substances was achieved. The methods of isolation and purification (Figure 6Figure 7 and Figure 8) of individual biologically active substances reflect the features of the object (extract), namely the matrix effect of the components present, the concentration of the main target substances, the presence of accompanying impurity components. Analysis of the results of studies on the isolation and purification of individual BAS obtained from extract samples allowed us to establish that the isolated target biologically active substances had a degree of purification of at least 95%, while after the first stage (Figure 6), it was only 50%. Callus cultures of *Ginkgo biloba* (L.) produced quercetin, kaempferol, luteolin, isorhamnetin, amentoflavone, bilobalide, ginkgolide A, ginkgolide B, and ginkgolide C, as well as extracts of ginkgo leaves, represented mainly by aglycones of flavonol glycosides, such as kaempferol, quercetin, aglycones, flavon glycosides, such as apigenin, isorhamnetin, etc, and terpenoids, such as ginkgolides A,B,C, and bilobalides [9].

During the work, the qualitative content of flavonoids was determined using a qualitative reaction [35], and the quantitative content was determined using column chromatography and HPLC. In the study [36], it was shown that flavonoids were determined by combining near-infrared spectroscopy with chemometry with qualitative and quantitative analysis of flavonoid concentrations in *Ginkgo biloba* extracts. Geng et al. [37] established a quantitative method of diffuse reflection spectroscopy in the near-infrared region for the simultaneous determination of three flavonol aglycones in ginkgo biloba extracts. Shi et al. [38] determined flavonoids based on the method of colorimetric analysis with minor modifications. It was found that chromatography was used to obtain 66.58 mg/total amount of biologically active substances of flavonoids of *Ginkgo biloba* callus culture on average (Table 3). The data obtained differ from the data presented in the study [36], in which the yield of flavonoids averaged 28.55 mg/100 g of *Ginkgo biloba* leaves (L.). Thus, it can be stated that the quantitative determination of flavonoids by the chromatographic method makes it possible to more fully and accurately determine their content in comparison with the method of diffuse reflection spectroscopy in the near-infrared region and photocolorimetry [36].

Currently, research is aimed at developing technologies for obtaining extracts from ginkgo leaves [39,40]. Factors determining the advantage of the approaches are the maximum degree of extraction of the target components, low extraction costs, and the amount of ballast substances [41]. The emerging trends in the use of plant materials as extractants, namely ionic solutions, eutectic mixtures, etc., have an undoubted advantage [39,42]. The use of enzyme pretreatment of the sample also contributes to an increase in the yield of extractive substances. However, all these approaches are aimed at increasing the total yield of extractive substances, whereas a small number of publications are devoted to the isolation of high-purity components with a high potential for therapeutic safety [43,44,45]. The extraction options proposed in our study (Figure 6) and sorption chromatographic purification options (liquid chromatography was carried out in a TSX Series chromatographic refrigerator powered by V-Drive thermos scientific at a temperature of 10 °C) (Figure 7 and Figure 8) provide the best release of the target component from accompanying impurities in relation to the extract of the *Ginkgo* callus culture. It should be noted that the use of sorbents and similar technological techniques is used in the field of phytochemistry and analysis for extracting components of plant origin [39,40]. In [33], for example, a polyamide sorbent was used, which allowed researchers to obtain purified flavonoid fractions with an increase in the total content of up to 50%. However, in our study, variants of preparative accumulation of target components have been developed, taking into account the matrix effect of *Ginkgo* calluses with a degree of extraction of at least 50% from extracts of callus cultures and a degree of purification of at least 95% according to HPLC data.

In the preparative mode, when separating mixtures and isolating individual components, the number of components contained in the extract, as well as related substances, should be taken into account. In the separation process, a critical role is played by the structure of the target component (lipophilic–hydrophilic properties, including van der Waals interactions) and the spatial arrangement of substituents. Thus, flavonoids with three hydroxyl groups and methyl esters are most effectively separated under polyamide conditions. In turn, glycosidized forms of flavonoids are effectively separated on cellulose and silica gel [46]. Of particular note are the properties of Sephadex LH-20, with its universal lipophilic–hydrophilic properties and high mobile phase stability at a wide range of pH values, as well as inertia to organic solvents [47]. This property of the sorbent is actively used in phytochemical analysis and for purification (post-purification) of pharmaceutical substances in the column chromatography mode.

Under the conditions of using various sorbents, the yield of target products increased from 24% to 60% [48,49]. Methods of purification of *Ginkgo biloba* extracts are still at the stage of scientific formation and comprehensive study (high consumption of organic solvent, environmental pollution, reduced activity of the target component and, often, low purity of the resulting product). The considered methods, as well as other experimental works, are aimed at obtaining total extracts of BAS of a certain class based on a certain polarity [33].

## 4. Materials and Methods

### 4.1. Seed Culture for Callus Induction

Callus cultures of ginkgo seeds *Ginkgo biloba* (L.), grown in vitro, obtained at the early stages of the study were the object of the study. To obtain callus cell cultures, seeds of *Ginkgo biloba* were used as the starting material.

To obtain a sterile *Ginkgo biloba* material, seeds impregnated with 96% ethyl alcohol were burned three times in the flame of an alcohol lamp with fire extinguishment after 4–5 s, after which the hard shell was removed and sterilized in 70% ethyl alcohol for 1 min and 0.1% sulema solution for 7 min. After sterilization, the material was washed three times for 20 min in distilled sterile water. After sterilization, to obtain sterile seedlings, the seeds were planted on agarized media in Petri dishes with a diameter of 60 mm and 90 mm, as well as in jars with ventilated lids [50]. 

Here, MS medium, in combination with growth regulators 2,4-D (2 mg/L) and kinetin (0.10 mg/L), was used for callus induction.

### 4.2. Extraction of Biologically Active Components

In order to obtain extracts, rational parameters were selected for extracting a complex of biologically active substances with potential geroprotective properties from the biomass of *Ginkgo* callus cultures. Ethyl alcohol was used as an extractant. The dried callus was crushed in a mill and sieved through a sieve with a hole size of 1 mm. Fine powder of the studied plant (dried callus 3.0 g) was extracted in 260 mL of ethyl alcohol of various concentrations (30%, 50%, and 70%) under static conditions to obtain BAS. The extraction of plant material was carried out in a water bath with a reverse refrigerator. The extraction frequency is two. The extractant concentration (C, %), extraction temperature (t, °C), and extraction exposure (τ, h) (Figure 1) were used as independent variables. Extraction parameters were selected based on the results of the study [51]. The parameters included the concentration of the extractant (0, 10, 20, 30, 40, 60, 80, and 100%, weight/weight), extraction temperature (25, 30, 35, 40, 45, 50, 55, and 60 °C), the ratio between the volume of the extractant and the powder of Ginkgo biloba leaves by weight (7.5:1, 10:1, 12.5:1, 15:1, 20:1, 30:1, and 50:1, mL·g^−1^), and the extraction time (5, 10, 15, 20, 25, 30, and 40 min). The choice of extraction parameters is confirmed by the study [52], in which temperature (from 35 to 65 °C), the concentration of the ethanol extractant (from 39 to 90%), extraction time (from 1.5 to 2.0 h), and pressure (from 18 to 35 MPa), were selected as extraction parameters.

### 4.3. Method of Isolation of Biologically Active Substances

The isolation of individual biologically active substances from the extract of *Ginkgo biloba* includes the following steps, shown in Figure 6. Initially, the extract was filtered through filters (cellulose), diluted with water, and then the samples of the mixture were kept for 48 h at a temperature of + 4 °C (filtration of lipid precipitation). Then, the extract samples were concentrated in a vacuum in the presence of sodium chloride (up to 10% of the salt content in the solution), and then removed by decanting resinous substances from the remainder of the transparent solution. Purification from lipophilic substances was carried out by liquid–liquid extraction with *n*-heptane, and amounts of terpenolactones were isolated. After the three-time extraction of the aqueous phase with *n*-butanol, three phases were combined into one *n*-butanol phase, which was then concentrated under vacuum to a dry residue. At the next step, the residues were dissolved in an aqueous alcohol solution. The liquid–liquid extraction phases were purified with ethyl acetate, followed by washing with a sodium chloride solution and evaporation to a dry residue of the ethyl acetate-washed phase.

### 4.4. Method of Purification of Biologically Active Compounds

Purification from lipophilic substances was as follows: liquid–liquid extraction with *n*-heptane, followed by isolation of the sum of terpene lactones; the aqueous phase is extracted three times with n-butanol, and all three phases are combined into one *n*-butanol phase, which is then concentrated under vacuum to a dry residue; dissolution of the residue in an aqueous alcohol solution then occurs. Purification of the phase by liquid–liquid extraction with ethyl acetate takes place, followed by washing of the ethyl acetate phase obtained at stage 6 with a sodium chloride solution, before evaporation to dry residue of the washed ethyl acetate phase. 

As a result of column chromatography, fractions of the following individual biologically active substances are obtained: bilobalide, ginkgolide A, ginkgolide B, and ginkgolide C (Figure 7).

Final purification was carried out as follows. The dissolution of the dry residue was carried out in acetone containing 40 wt. % water, with cooling of the resulting suspension to 10 °C for one hour, followed by filtration.

Flavone glycosides were chromatographed on polyamide (Sigma-Aldrich, Berlin, Germany) and packed in a 5.3 × 250 mm chromatographic column on a BioLogic low-pressure chromatograph (BioRad, Hercules, CA, USA) using the following gradient eluting mixtures: chloroform–methanol (100:0 → 60:40), followed by water–ethanol (100:0 → 0:100). 

For the complete separation of the components and their purification, Lachema silica gel rechromatography with a particle size of 40/100 µm proved to be effective. A mixture of chloroform and petroleum ether was used as the mobile phase in the ratio of 30:70, followed by recrystallization of substances. This procedure made it possible to isolate the following flavonoids: quercetin, kaempferol, luteolin, isorhamnetin, and amentoflavone.

The use of the fraction purification scheme (Figure 7 and Figure 8) made it possible to obtain individual biologically active substances from the extracts of ginkgo biloba with a degree of purification of individual BAS of at least 95%. 

### 4.5. High Performance Chromatography Method

The Prominence LC-20 series (Shimadzu) chromatograph with a diode-matrix detector SPD20 M, autosampler SIL-20AS, pumps LC-20AD, degasser DGU-20 As, column thermostat CTO-20A, detector with diode matrix SPD-M20A, fluorometric detector, and CBM-20A system controller was used to analyze the component content of aqueous–alcoholic extracts of *Gingko biloba*. In this study, LC (Shimadzu, Corporation) software, version 5.23 SP1, was used. A Phenomenex Gemini C18 column (110 Å, 5 µm, 4.6 mm×250 mm) was used as a stationary phase, with a mobile phase consisting of deionized water–acetonitrile with the addition of formic acid. The following mixtures were used: water–acetonitrile 95:5 + 0.1% formic acid (eluent A) and acetonitrile–water (95:5) + 0.1% formic acid (eluent B). The flow rate was 0.8 mL/min, at a temperature of 40 °C. The separation was carried out in the gradient elution mode according to [53]. Injection volumes were 20 µL, in five repetitions. Relative retention times were determined using flavonoid and terpene lactone standards (Acros, J and K, Aldrich-Sigma). The quantitative determination of the isolated components was performed under the HPLC method conditions. A diode-matrix detector with a wavelength range of 190–750 nm was used for this research.

### 4.6. HRMS Spectrometry

Mass spectra were recovered using a Bruker Amazon speed spectrometer (ionization by electrospray, positive ion detection).

### 4.7. Low-Pressure Column Chromatography Method

Low-pressure column chromatography was performed on a BioLogic chromatograph (BioRad, Hercules, CA, USA), and concentration to dryness of the supernatant (n-heptane phase) containing terpene lactones was performed on a silica gel 60 (Macherey-Nagel, Duren, Germany) fraction of 0.2–0.5 mm. A mixture of water–methanol–tetrahydrofuran was used as the mobile phase in the corresponding ratios of 75:20:10, and a column was used with a size of 30 × 300 mm.

Chromatography of flavon glycosides was performed on polyamide (Sigma-Aldrich, Germany) packed in a 5.3 × 250 mm chromatographic column on a BioLogic low-pressure chromatograph (BioRad, Hercules, CA, USA) using gradient eluting mixtures, as follows: chloroform–methanol (100:0 → 60:40), then water–ethanol (100:0 → 0:100). 

For complete separation of the components and their purification, silica gel rechromatography on a silica gel 60 (Macherey-Nagel, Duren, Germany) fraction of 0.2–0.5 mm was used, using the following eluent mixture: chloroform–petroleum ether in ratios of 30:70, followed by recrystallization of the substances [48,54,55,56].

### 4.8. Spectrophotometry of Samples 

The spectral characteristics of the total extracts, as well as individual isolated components (UV spectra) were recorded on a spectrophotometer (OKB Spectrum LLC, St. Petersburg, Russia) in the wavelength range of 190–600 nm with a resolution of 0.5 nm in liquid cuvettes with an optical path length of 10 mm. Both pure components and with the addition of reagents were photometrized to reveal the general and specific properties of flavonoid compounds and differential spectra after the addition of specific reagents (AlCI_3_/HCI, NaOMe, NaOAc, and NaOAc/H_3_BO_3_) [57,58]. 

### 4.9. IR Spectrometry

Infrared spectra were obtained from a disk with potassium bromide in the range of 4000–400 cm^−1^, with a resolution of 4 cm^−1^, and 50 accumulation cycles using a Fourier spectrometer FSM–2202 [59]. 

### 4.10. Sample Preparation

Acid hydrolysis was carried out in the following way: 2 mL of the extract was placed in a conical flask with a capacity of 100 mL; 20 mL of MeOH and 2N HCI (1:1) were added, treated with ultrasound for 5 min, and hydrolysis was carried out in a boiling water bath with a vapor condensation device for 20 min. The solutions was then evaporated under vacuum to a dry residue and dissolved in 2 mL of the mobile phase.

The relative standard deviation was calculated for the peak areas of quercetin (Sigma-Aldrich, Berlin, Germany) and kaempferol (Sigma-Aldrich, Berlin, Germany) by five chromatograms, and the deviation did not exceed 0.5%. The efficiency of the chromatographic column calculated according to the standards of quercetin and kaempferol amounted to more than 10,000 theoretical plates [60,61].

### 4.11. Statistical Analysis Methods

Statistical processing was performed using Excel (2019, Microsoft, Redmond, Washington, DC, USA) and Statistica 10.0 (StatSoft Inc., 2007, Tesla, WV, USA). All experiments were carried out in triplicate. Data are presented as the median ± standard deviation. The Kruskal–Wallis test was used to compare the medians of the samples (significant differences at *p* < 0.05). For intergroup comparisons, the Mann–Whitney U test was used with the Bonferroni correction (significant differences at *p* < 0.01). To check for the presence of a correlation between extraction methods and quantitative indicators of extracts, the Spearman’s rank correlation coefficient was used (significant differences at *p* < 0.05).

## 5. Conclusions

The development of technology for the isolation and purification of target biologically active components using callus cultures is of undoubted interest for the pharmaceutical and food industries. The need for extracts and purified biologically active substances of *Ginkgo biloba* increases every year. The main components produced by the *Ginkgo biloba* callus culture were flavonoids (quercetin, kaempferol, luteolin, isorhamnetin, and amentoflavone) and triterpenoids (bilobalide, ginkgolide A, ginkgolide B, and ginkgolide C). With a selected and experimentally justified extraction mode (the extractant used was 70% ethanol, temperature 50 °C, and exposure time 6 h), the target extract was obtained. In our study, it was possible to optimally select the sequence of application of sorbing materials, which contributed to the effective isolation of target molecules. The developed methods of isolation of individual components can be scaled and used in the production process of isolation and preparative accumulation of target substances produced by *Ginkgo biloba callus* cultures. Therefore, the extraction and sorption–chromatographic approaches proposed in this work can be used in laboratory practices and industrial production in the development and production of pharmaceutical substances and functional products.

## Figures and Tables

**Table 1:** Optical density of extracts of *Ginkgo biloba* callus cultures.

Extraction Mode	Temperature, °C	Volume Fraction of Ethanol in the Extractant, %	Optical Density		
			Duration of Extraction, h		
№			2	4	6
1	30	30	0.2300 ± 0.0025	0.2090 ± 0.0036	0.2230 ± 0.0030
2	30	50	0.1410 ± 0.0010	0.2450 ± 0.0035	0.2310 ± 0.0010
3	30	70	0.1830 ± 0.0022	0.2060 ± 0.0010	0.3100 ± 0.0015
4	50	30	0.2530 ± 0.0030	0.2530 ± 0.0030	0.2680 ± 0.0033
5	50	50	0.2200 ± 0.0015	0.2250 ± 0.0010	0.2860 ± 0.0022
6	50	70	0.2480 ± 0.0028	0.2790 ± 0.0019	0.3950 ± 0.0031
7	70	30	0.2100 ± 0.0010	0.2630 ± 0.0036	0.3020 ± 0.0041
8	70	50	0.2430 ± 0.0030	0.2850 ± 0.0020	0.2790 ± 0.0029
9	70	70	0.2450 ± 0.0034	0.2560 ± 0.0028	0.2590 ± 0.0010

Data presented as a mean ± SD (*n* = 3). All values in columns/row do not differ significantly (*p* > 0.05).

**Table 2:** The component composition of *Ginkgo biloba* extract according to HPLC data.

Peak No.	Retention Time, min	Component Name	Quantitative Content, μg/mL
1	4.76 ± 0.80	Ginkgolide A	-
2	5.16 ± 0.80	Ginkgolide B	-
3	6.60 ± 0.80	Bilobalide A	5.93 ± 0.27
4	6.92 ± 0.80	Ginkgolide C	5.23 ± 0.27
5	8.05 ± 0.80	Quercetin	11.40 ± 0.76
6	9.03 ± 0.80	Ginkgetin	0.64 ± 0.05
7	9.93 ± 0.80	Isorhamnetin	17.92 ± 0.93
8	13.72 ± 0.80	Luteolin	25.63 ± 0.86
9	17.49 ± 0.80	Kaempferol	4.63 ± 0.50
10	23.82 ± 0.80	Amentoflavone	7.91 ± 0.50

The data are expressed as mean ± standard deviation (*n* = 3).

**Table 3:** Mass yield of extractives substances.

Peak No.	Component Name	Substance Content in Extract *, %	Purity Degree after Purification According to HPLC *, %	Substance Yield *, Mg/Total Amount of Biologically Active Substances
1	Ginkgolide A	-	95.3	14.0
2	Ginkgolide B	-	95.1	75.0
3	Bilobalide A	6.14	96.3	43.4
4	Ginkgolide C	5.42	95.6	48.3
5	Quercetin	11.81	99.8	93.4
6	Ginkgetin	0.66	97.1	3.7
7	Isorhamnetin	18.57	99.5	110.0
8	Luteolin	26.56	99.4	189.3
9	Kaempferol	4.79	98.7	38.4
10	Amentoflavone	8.19	96.3	50.3

* Average of three measurements.

**Table 4:** IR characteristics of ginkgolide A.

Wave Number, cm^−1^	Note
3422	ν (O-H) of the intramolecular H bonds
2964	ν (as) C-H в CH_3_
2922	ν (as) CH_2_
2851	ν (s) CH_2_
1735	Lact.
1631	ν (C-O-C)
1199	ν (C–C( = O)–O)
1157	ν (C–O–C)
1112	ν (C–OH)
1043	ν (O-C-C)
903	tBut

**Table 5:** IR characteristics of ginkgolide B.

Wave Number, cm^−1^	Note
3430	ν (O-H) of the intramolecular H bonds
2954	ν(as) C-H в CH_3_
2921	ν (as) CH_2_
2850	ν (s) CH_2_
1737	Lact.
1718	ν (C = O)
1631	ν (C-O-C)
1571	ν (C–O–C)
1114	ν (C–OH)
1043	ν (O-C-C)

**Table 6:** IR characteristics of ginkgolide C.

Wave Number, cm^−1^	Note
3435	ν (O-H) of the intramolecular H bonds
2955	ν (as) C-H в CH_3_
2921	ν (as) CH_2_
2850	ν (s) CH_2_
1737	Lact.
1716	ν (C = O)
1631	ν (C-O-C)
1107	ν (-C( = O)-C
1043	ν (O-C-C)
849	ν (C-O-C) lact.
805	ν (-CH_2_-)

**Table 7:** IR characteristics of bilobalide.

Wave Number, cm^−1^	Note
3405	ν (O-H) of the intramolecular H bonds
2969	ν (as) C-H в CH_3_
2924	ν (as) CH_2_
2853	ν (s) CH_2_
1785	Lact.
1628	ν (C = O)
1380	δ(O-H)
1158	ν (C–O–C)
902	δ( = C-H)

## Data Availability

Not applicable.

## Sample Availability

Samples of the compounds are not available from the authors.
